# Computational and Experimental Mechanical Modelling of a Composite Grouted Splice Sleeve Connector System

**DOI:** 10.3390/ma11020306

**Published:** 2018-02-20

**Authors:** Zhiping Kuang, Guanyu Zheng

**Affiliations:** Department of Building Engineering, College of Civil Engineering, Tongji University, Shanghai 200092, China

**Keywords:** composite, connection, grouted splice sleeve, finite element, material nonlinearity

## Abstract

Owing to its controllable tolerance, simple operation and no need for welding at construction site, the composite system involving grouted cement material, steel material and ductile iron material is widely used as grouted splice sleeve (GSS) connector for connecting precast concrete structures. However, the current design recommendations for such a composite connection system do not accurately account for its material nonlinearity behavior. In the present study, a three-dimensional nonlinear finite element model of a GSS connector is developed by considering the nonlinear material behavior of each component to fully investigate its mechanical performance under axial tension. To validate the proposed computational model and demonstrate the nonlinear response of the GSS connector, the pullout experimental test of two engineering specimens is carried out under monotonic tensile load, and a good agreement between the numerical and experimental test results is observed. Then, the sensitivity analysis of some controlling material properties and geometrical parameters is performed using the validated computational model to further understand the performance of such a composite structure in load carrying capacity and ductility of the connections to meet the rapid engineering applications of precast concrete structures.

## 1. Introduction

Compared to the traditional cast-in-place concrete structures, the precast concrete structures [1] are usually manufactured in a controlled environment, i.e., plants, and then are installed at the construction site through certain connections. Hence, the precast concrete structures can have better concrete quality and help to reduce the cost of labor and increase speed of construction. Due to these advantages, the precast concrete structures have received much attention in recent years.

**Figure 1:**
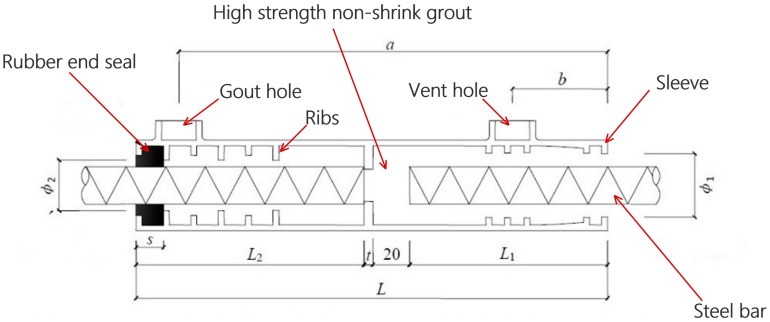
Detailing of a typical engineering grouted splice sleeve connector.

In previous engineering practices, the popular connections of reinforcing bars in precast elements are operated through lapped splices, welded splices and bolted connections [2]. Since the rigid grouted splices were invented [3], they have been used as the preferred technology to splice reinforcing steel bars particularly in precast concrete elements, due to its controllable tolerance, simple operation and no need for welding at the construction site. As a typical precast concrete mechanical connector system, the grouted splice sleeve (GSS) connection mainly has the advantages of bond-slip resistance of grout and mechanical gripping of reinforcement bars to facilitate the coupling of steel reinforcement bars and provide tensile and compressed resistance [4,5]. Figure 1 illustrates a typical GSS connector used in engineering. In this connector system, the reinforcement bars from precast elements are inserted into a hollow cylinder (sleeve) from both ends and then high-strength cement grout is filled into the sleeve at both sleeve ends as a load transferring medium and bonding material to splice reinforcement bars. Therefore, the application of GSS connector can significantly reduce the required rebar lap length and simultaneously maintains continuity of reinforcement between precast elements and structural integrity. Some studies have been conducted to investigate the mechanical performance of the GSS connector. For example, Arditi et al. investigated the factors affecting the use of precast concrete elements [6]. Zhao et al. analyzed the influence of elevated temperatures on the load carrying capacity of prestressed grout sleeve connections [7]. Ling et al. tested the tensile capacity of grouted splices connected by a welded bar sleeve and tapered head sleeve [8]. Lin and Wu studied the mechanical performance and the related stress-strain relationship for a GSS connection [9]. Apart from the widely used steel sleeves, the splicing techniques with fiber-reinforced polymer sleeve were also studied and the results revealed that splicing may be improved by introducing a composite sleeve [10]. Moreover, regarding seismic response of precast concrete structures connected by GSS joints, Belleri and Riva studied the suitability of grouted sleeve connections as column-to-foundation connections for precast concrete structures in seismic regions [11]. Popa et al. performed experimental testing on emulative GSS connections for precast columns in seismic region and concluded that the tested specimens have a similar hysteretic response and energy dissipation capacity to the reference ones for each level of applied axial force [12]. More recently, the seismic behavior of GSS connectors was analyzed respectively for precast concrete bridge columns [13] and shear walls [14].

However, most of the literature mentioned above focused on the experimental testing of the GSS connector. As an alternative to experimental methods, the numerical methods such as the finite element method [15,16,17,18], the hybrid finite element method [19,20,21,22] and the boundary element method [23,24,25] can be employed to quantitatively simulate the mechanical behavior of complex precast concrete structures in a more flexible and cheaper way. However, despite its advantages, to the authors’ knowledge, few research efforts have addressed the splicing mechanism in the GSS connector system through the nonlinear numerical simulation [26]. Moreover, the current design recommendations [27,28,29] for such a connection do not accurately account for the nonlinear effect of materials in it. Thus, it is necessary to perform the highly-efficient nonlinear mechanical analysis of GSS connectors to further understand their mechanical response to meet rapid engineering applications of precast concrete structures.

In the present paper, providing a more flexible and cheaper tool for the highly efficient analysis of GSS connector is the first objective. To do so, a three-dimensional (3D) nonlinear finite element model of GSS connection is established by introducing the nonlinear stress-strain relations respectively for the high-strength non-shrink grout material, the steel reinforcement bars and the sleeve. The second objective of this paper is to carry out the corresponding experimental test to show quantitative experimental evidence of the behavior of a typical GSS connector under tension so that the present numerical model can be validated. Besides, it is well known that the strength of GSS connector mainly relies on the bonding mechanism between the grout and the reinforcement bars. The geometry and material properties of the sleeve and the reinforcement bars and the compressive strength of the grout all contribute to the bonding strength. Hence, the third objective of this study is to investigate the effects of some controlling parameters on the mechanical performance of a GSS connector through numerical analysis of 48 different connectors.

The paper is organized as follows. Section 2 describes the geometrical and structural features of a real GSS connector, and then the experimental program is simply described. In Section 3, the nonlinear finite element model is established by introducing the material nonlinearity of structural components. In Section 4, related results are discussed. Finally, some conclusions are summarized in Section 5.

## 2. Experimental Program

In this section, a typical engineering grouted splice sleeve connector shown in Figure 1 is taken into consideration as a testing specimen and the related dimensions of the sleeve are presented in Table 1 for reference. In Figure 1 and Table 1, *L* is the sleeve length, *t* is the approximated wall thickness of the sleeve and the middle separate plate, $\phi_{1}$ is the diameter of the wide end, $\phi_{2}$ is the diameter of the narrow end, *a* is the distance from the wide end to the grout hole, *b* is the distance from the wide end to the vent hole, *s* is the thickness of the rubber end seal, *t* is the thickness of middle separate plate, and $L_{1}$ and $L_{2}$ are the embedded lengths of steel bars, respectively.

The sleeve with circular hollow section has an average wall thickness of 5 mm, and is generally made of cast of ductile iron, whose ultimate tensile strength (UTS) is usually more than 550 MPa. The steel bars are spliced at the embedded lengths as reinforcement bars, and there is approximately 20 mm distance between them. Also, the steel bars should be aligned without eccentricity to avoid undesired stress in the connector under tension. In the experiment, two types of steel bars, H400-20 and H500-20, are taken into consideration and their nominal diameter is 20 mm, but their yield strength is 400 MPa and 500 MPa, respectively. Besides, the commercial available high-strength and non-shrink grout with the nominal compressive strength of 70 MPa at day 28 is used as the bonding material between the sleeve and the reinforcement steel bars. It is usually mixed into a pour-able state according to the proportion given by the manufacturer so that it can be poured easily into the sleeve through the grout hole by a grout pump. If the grout outflows from the vent hole, pumping is stopped and the sleeve is assumed to be fully filled with the grout material. Then the vent and grout holes are sealed for protection. Obviously, the bond performance of the spliced steel bars is improved as the expansion of the grout material is confined by the sleeve. Additionally, one observes from Figure 1 that the small ribs on the steel bars and on the wall of sleeve interlock with the grout to prevent the grout from slipping out of the sleeve and simultaneously generate excessive confinement stress to improve the bond inside the sleeve. However, we have to note that the existence of ribs leads to the development of internal micro-cracks during the tension of the connector, due to stress concentration. As a result, the tensile stiffness of the connector will have an understandable but insignificant decrease [2,8].

**Figure 2:**
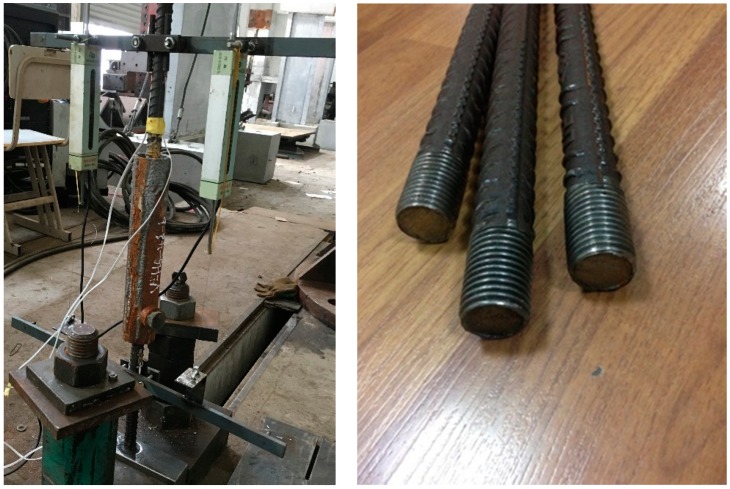
Test setup for specimens under incremental tensile load.

To investigate the pullout behavior of the grouted connections, an experimental program is designed to measure the mechanical response of two specimens with H400-20 and H500-20 reinforcing steel bars under tensile conditions. Figure 2 depicts the practical test setup and the real configuration of steel bars. In the fabrication of each connector specimen, the sleeve is firstly placed vertically by means of a fixture, then the bars are carefully inserted into the sleeve from the two ends to make the two bars align without eccentricity, if possible. Subsequently, the grouting operation can be performed. After grouting, the grouted splice specimen is removed from the fixture after 24 h and then is horizontally placed at room temperature for about 28 days until testing. Finally, the grouted specimen is amounted in a 500kN servo-hydraulic material testing machine for tensile loading. All the specimens are loaded with a tensile speed of 0.1mm/s, which is a displacement controlled mode to capture the ultimate load of the connection. For each test, the recorded data include loading forces and averaged splice elongations measured by two deformation gauges which are respectively mounted on the two sides of the spliced bars at about five bar diameters from the surface of the grout. The loading forces are recorded automatically by a data acquisition device of the actuator. As a result, the related curves of load and splice deformation can be obtained, which are used to validate the present nonlinear finite element model in Section 4.

## 3. Nonlinear Finite Element Model

**Figure 3:**
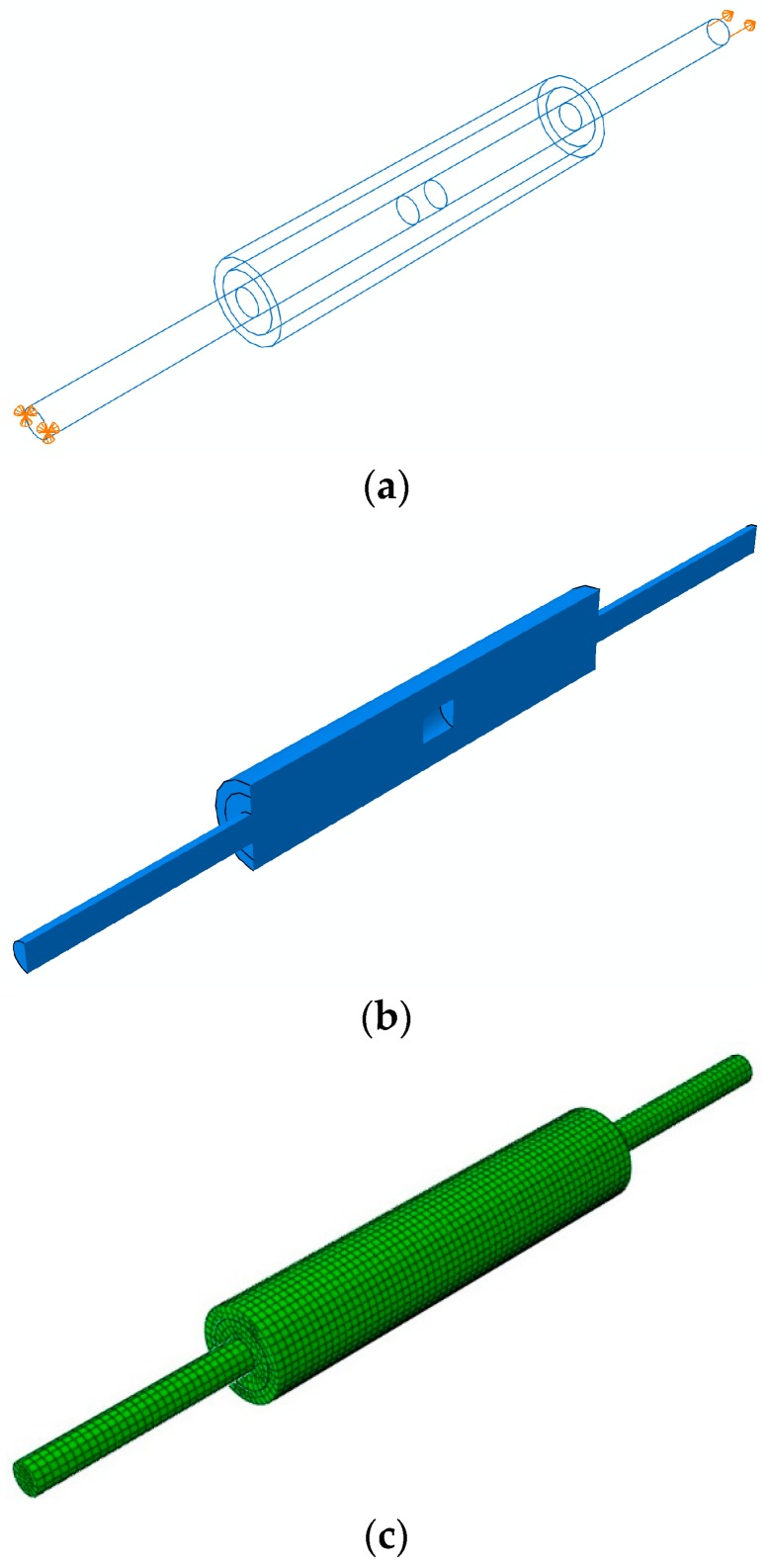
Illustrations of (**a**) the full computational model with cell boundaries and applied boundary conditions; (**b**) cut view of the computational model and (**c**) the finite element meshing.

Generally, the experiments are time-consuming and involve high cost. As an alternative, the numerical method, i.e., finite element method used in the study, provides a more flexible and cheaper way to solve the practical problems [15]. Here, the nonlinear finite element model is developed for the mechanical analysis of a series of GSS connectors by introducing nonlinear material constitutive models [30]. For the sake of simplification, the ribs of the reinforcement bars and the sleeve, the grout and vent holes, the end sealing rubber and the wall thickness change of the sleeve displayed in Figure 1 are neglected in the present computational model, as depicted in Figure 3, so that the computational model of the GSS connector system only consists of the sleeve, the reinforcement bars and the grout. In addition, to simplify the computational analysis, the perfect bonding connection is assumed between adjacent structural components, which is also assumed for the analytical model developed in [2,8], due to the so insignificant slip and the good confinement in the sleeve. Certainly, one can introduce more complex bond-slip interfacial models for engineering analysis, according to one’s need [31,32,33].

In the above computational model of GSS connector system under consideration, three different materials are involved. To deal with material nonlinearity, the following properties of all materials are described.

### 3.1. Grout

#### 3.1.1. Compressive Stress-Strain Relation

**Figure 4:**
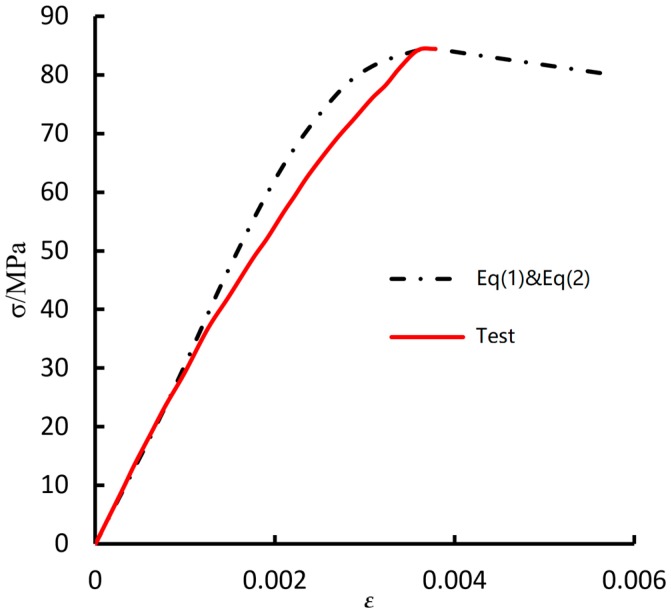
Uniaxial compressive stress-strain relationship of grout.

The experimental data of the stress-strain test can be used to model the compressive behavior of grout material. Figure 4 shows the stress-strain curve of grout material obtained by the uniaxial compression experiment (see solid line). In order to approximate the variation of stress and strain under the uniaxial stress state given in Figure 4, in this analysis, the mortar constitutive model [34] is modified to provide better fitting to the test curve. In the modified constitutive model, the stress-strain relation includes two stages: the ascending stage and the descending stage. For the ascending stage, it is assumed that

(1) $$\sigma =\frac{\varepsilon \varepsilon_{0}f_{c}}{0.85\varepsilon^{2}-0.7\varepsilon \varepsilon_{0}+0.85\varepsilon_{0}^{2}}$$

and for the descending stage we assume

(2) $$\sigma =(1.1-0.1\frac{\varepsilon }{\varepsilon_{0}})f_{c}$$

where *σ* is the stress component and *ε* is the strain component. *f_c_* is the peak stress measured by the grout’s compression test, and *ε_0_* is the corresponding strain.

It is observed from Figure 4 that the assumed ascending Equation is very close to the experimental curve of grout material. Therefore, Equations (1) and (2) will be used to describe the compressive behavior of the grout material.

#### 3.1.2. Tensile Stress-Strain Relation

**Figure 5:**
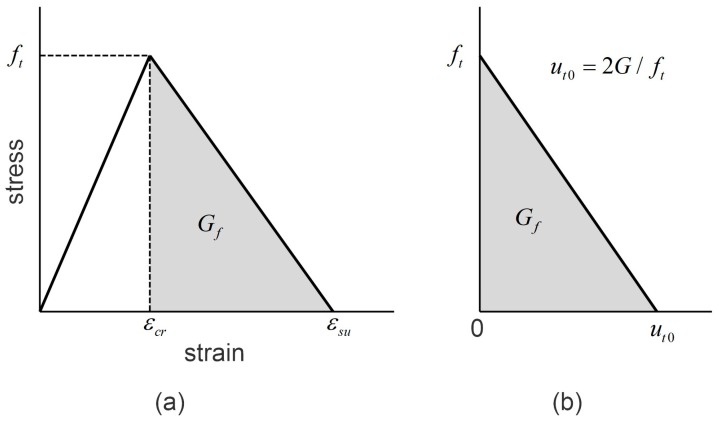
(**a**) Uniaxial tensile stress-strain relation of the grout; (**b**) Softening modeling of the grout in tension.

To represent the stress-strain relation of uncracked grout in tension, the bilinear constitutive model shown in Figure 5a is employed [28]. In Figure 5a, the linear portion of a tensile stress-strain response terminates when the stress reaches its ultimate value *f_t_* (tensile strength). The corresponding cracking strain is *ε_cr_*. It is assumed that the elasticity modulus for tension and compression of grout is the same at this linear stage. Subsequently, the material softening occurs, and the stress begins to linearly decrease as the strain increases. The tensile response terminates at the ultimate tensile strain*ε_su_*. Figure 5b represents the related stress-deformation curve for the strain softening region, and it is obvious that the area in Figure 5b can be used to represent the fracture energy *G_f_*. In Figure 5b, the horizontal axis stands for the crack width, whose maximum value can be determined through $u_{t0}=l_{c}(\varepsilon_{su}-\varepsilon_{cr})$, where *l_c_* is the strain gauge length. According to European standard MC90 [27], the fracture energy of grout can be defined as

(3) $$G_{f}=\alpha {\left(\frac{f_{c}}{10}\right)}^{0.7}$$

where *f_c_* is the compressive strength of grout, and its value is from the cubic compressive test. *α* denotes the modified coefficient. For the grout material, which usually includes large number of tiny particles, we can approximately take α = 0.02Nmm/mm^2^ for the actual analysis [27].

### 3.2. Steel Bars

**Figure 6:**
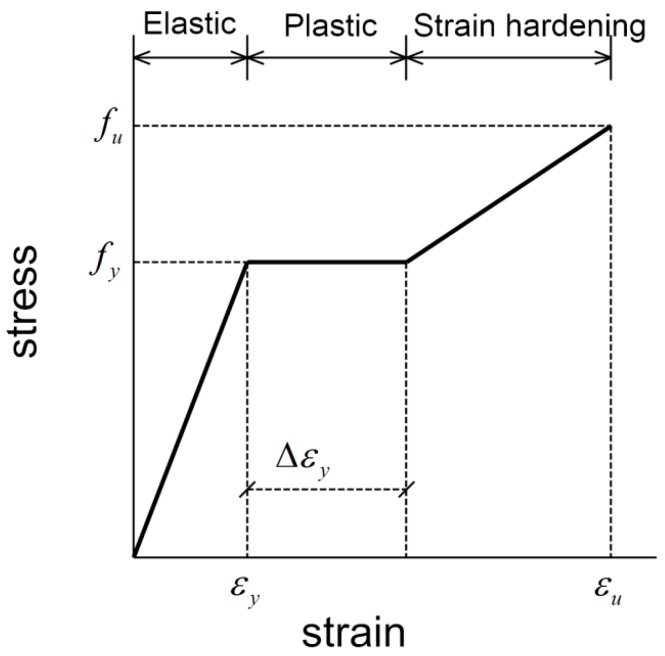
Idealized trilinear stress-strain diagram for steel reinforcements.

As a classic simplification, the actual constitutive diagram of steel material in tension can, in finite element computation, be replaced by an idealized characteristic trilinear diagram [1,30], as given in Figure 6. The steel is assumed to have a linear stress-strain relation until the yield stress *f_y_* is reached. After the yield stress, it is assumed that the stress in the steel remains constant as the strain increases. Then, the strain hardening occurs. In Figure 6, *f_y_* is the yield strength, Δε*_y_* is the length of yield plateau and *f_u_* is the ultimate tensile strength. These key parameters can be determined from the actual tensile experiment of sample. Furthermore, from the elastic region, the material elastic modulus *E* can be evaluated by *f_y_*/ε*_y_*. In the computation, the elastic modulus of all steel material is 2.00 × 10^5^ MPa. In addition, the Poisson’s ratio is supposed to be *ν* = 0.3.

### 3.3. Sleeve

For the ductile iron material used for the sleeve element, a simpler bilinear model is adopted by neglecting the strain hardening of the metal material given in Figure 6. This results in the elastic-perfectly plastic stress-strain model [30]. Therefore, the uniaxial stress-strain relation of the sleeve can be written as

(4) $$\sigma =\{\begin{array}{c} E_{s}\varepsilon_{s},\varepsilon_{s}\leq \varepsilon_{sy}\\ f_{ys},\varepsilon_{sy}\leq \varepsilon_{s}\leq \varepsilon_{su} \end{array}$$

where σ*_s_* is the yield stress corresponding to the yield strain ε*_s_*, *f_ys_* is the yield stress of sleeve, ε*_su_* is the ultimate tensile strain, and *E_s_* is the elastic modulus. Typically, we can take ε*_su_* = 0.2 and *E_s_* = 1.69 × 10^5^ MPa here. Besides, the Poisson’s ratio is assumed to be 0.275 for all ductile iron materials.

## 4. Results and Discussion

In practical analysis, the structured technique is employed to assume the element type for meshing different parts using the ABAQUS software. Because the grout, steel bars, and sleeve are modeled as solid parts, the eight-node brick elements (C3D8) implemented in ABAQUS with reduced integration technique are used for meshing each part, as shown in Figure 3. In total, 10,182 elements and 13,055 nodes are involved in this meshing scheme. During the meshing procedure, the bias technique with bias ratio 3 is used to obtain relative dense mesh around the interface of grout material and the steel bars.

Additionally, one ending surface of the steel bar is fixed and another ending surface is stretched by giving displacement conditions. Generally, the grouted splice sleeve connector fails in the bar yield fracture which has higher tensile capacity and degree of ductility than the welded bar sleeve connector in practice [8]. In the experiment, when the steel bar facture takes place, the test stops. The computation stops just when the steel bar yields.

### 4.1. Validation

Firstly, the connector with the QT600 sleeve is considered in the experiment and computation, which has the yield strength 370 MPa and ultimate tensile strength 600 MPa. The two grades of spliced steel bars (H400-20 and H500-20) with diameter 20 mm are tested, whose yield strength and ultimate tensile strength are 400 MPa and 540 MPa for H400-20, and 500 MPa and 630 MPa for H500-20, respectively. To investigate the convergence of the finite element simulation, the three meshes with 6264 elements, 10,182 elements and 21,248 elements, respectively are respectively employed to discretize the computational model with H400-20 steel bars. The corresponding maximum Mises stress results on the surface of the sleeve are 247.23 MPa, 222.69 MPa, and 221.81 MPa, respectively. Thus, considering the requirement of computational accuracy and efficiency, we use the moderate-dense mesh discretization, i.e., 10,182 elements, in the following study.

**Figure 7:**
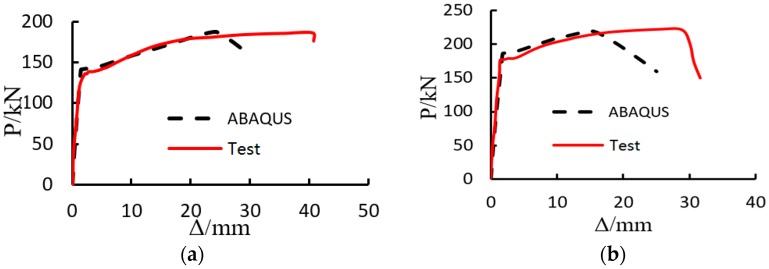
Load-displacement responses of the connectors for different steel bars. (a) H400-20; (b) H500-20.

Figure 7 indicates the numerical results of load-displacement responses for the H400-20 and H500-20 steel bars. Simultaneously, the experimental curve is provided in the figure to validate the present nonlinear finite element model. It is found that there is a good agreement between the numerical and experimental curves for both the H400-20 steel bars and the H500-20 steel bars, especially at the initial close-to-elastic stage and the yield and strengthening stage. Moreover, as the steel bar yields, obvious plastic response is observed, which causes significant elongation of the steel bar, and eventually causes the steel bar fracture as the tensile capacity is achieved. Thus, the present computational model is capable of simulating the mechanical performance of the GSS connector. However, it is also possible to observe that the numerical predictions give larger longitudinal displacement than the experimental curves in Figure 7. This can be attributed to (1) the use of approximated nonlinear material behavior in the computational model, (2) the difference of geometrical configuration of the computational model given in Figure 3 and the actual connector shown in Figure 1, and (3) the inevitable rebar eccentricity which can generate undesired stress in the connector to cause limited interfacial slip before or during bar yielding.

In addition, to investigate the load capacity of the GSS connector system, the results of yield load and ultimate load are tabulated in Table 2. In the table, $P_{y}^{\prime }$ and $P_{u}^{\prime }$ are the yield and ultimate loads from simulation, respectively. $P_{y}$ and $P_{u}$ are the corresponding results from the tensile test, respectively. Results in Table 2 show that the present nonlinear finite element model can provide highly accurate numerical solutions which are very close to the results from the tensile test. The maximum error between the numerical and experimental results is less than 4%. Such good agreement between numerical and experimental results validates the present computational model again. Besides, it is observed that both the yield load and the ultimate load of the connectors increase with the increase of yield strength of the steel bar, as expected. According to ACI-318 [28] and FIP-90 [27], the ultimate tensile strength of the connector, which can be calculated by the maximum load resistance divided by the sectional area of rebar, should be no less than 1.25 times the specific yield stress of the steel bar. In our experiment, this ratio is about 1.43 and 1.40 for H400-20 and H500-20, respectively.

### 4.2. Stress Results for Sleeve

After the validation of the present nonlinear finite element model, the tensile capacity of connectors under tensile load can be evaluated numerically by the quantitative analysis of some controlling parameters: (1) the diameter of steel bar, (2) the material parameters of steel bar, (3) the material parameters of sleeve. In total, three types of reinforcement bars are considered: H335, H400 and H500, whose yield strengths are 335MPa, 400MPa, 500MPa, and ultimate tensile strengths are 455 MPa, 540 MPa, 630 MPa, respectively. Their diameters are 12 mm, 18 mm, 20 mm and 22 mm, respectively. Moreover, the six types of sleeves, QT350, QT400, QT450, QT500, QT550 and QT600 are analyzed and their material properties are listed in Table 3.

**Figure 8:**
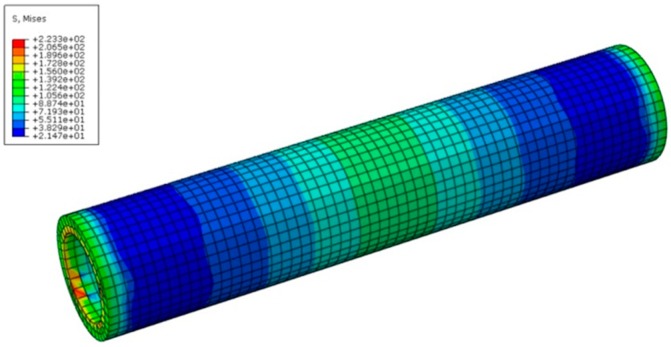
Misses stress distribution in the sleeve for the case of H400-20 and QT600.

We observe that the change of material properties of sleeve does not lead to significant difference of stress distribution in the sleeve practically. Figure 8 displays the variation of Mises stress in the sleeve part for the case of H400-20 and QT600. It is found that the maximum Mises stress locates at the inner surface of the sleeve, and its value is less than the sleeve yield strength 370 MPa. Thus, it is concluded that the sleeve is in the elastic state. Table 3 shows the results of maximum Mises stress in the sleeves with different material definitions for the three types of steel bars with specific diameters. It is found from Table 3 that, except for the steel bar H500 with diameter 20 mm, the yield in the sleeve made of ductile iron QT350 only takes place when the diameter of the reinforcement bars (H335 and H400) is no less than 20 mm. Thus, to avoid the sleeve facture in the GSS connector, it is suggested that the ultimate tensile strength of sleeve should be higher than 400 MPa, when the diameter of reinforcement bar is relatively large.

## 5. Conclusions

In this paper, the three-dimensional finite element analysis based on the nonlinear constitutive relations of material components in the composite GSS connectors is performed for predicting their nonlinear mechanical response under static tensile load, and the present computational model is validated through a comparison with the experimental results of two typical specimens with different steel bars. Subsequently, 48 different GSS connectors are analyzed by the present nonlinear finite element model with respect to various material and geometrical parameters. It can be concluded that:

1. The good agreement of the computational and experimental results shows that the present nonlinear finite element model can be applied for the analysis of GSS connector.
2. Both the yield load and the ultimate load of the GSS connector increase with the increase of the yield strength of the steel bar.
3. Except for the steel bar HR500 with diameter 20 mm, only the QT350 sleeve can reach plastic stress state when the diameter of reinforcement bar is no less than 20 mm.
4. To avoid the sleeve facture in the GSS connector, it is better that the ultimate tensile strength of the sleeve should be more than 400 MPa, when the diameter of reinforcement bar is relatively large.
5. With the nonlinear stress-strain relations introduced for various material components, the proposed finite element model can be used for the mechanical analysis of other connections such as Lap-spliced connectors and tapered sleeve connectors.

## Figures and Tables

**Table 1:** Details of dimensions of the connection (unit: mm).

Type	*L*	*Φ_1_*	*Φ_2_*	*a*	*b*	*s*	*t*	*L_1_*	*L_2_*
5VSA	220	31	20	47	189	17	5	94	101

**Table 2:** Results of yield and ultimate loads for the grouted splice sleeve (GSS) connectors.

Steel Bar	Yield Load (kN)		$\frac{P_{y}^{\prime }}{P_{y}}$	Ultimate Load (kN)		$\frac{P_{u}^{\prime }}{P_{u}}$
	$P_{y}^{\prime }$	$P_{y}$		$P_{u}^{\prime }$	$P_{u}$	
H400-20	133	138	0.96	179	187	0.96
H500-20	170	178	0.96	220	222	0.99

**Table 3:** Results of maximum Mises stress in the sleeve.

Reinforcement bar	Sleeve	Yield strength/ Ultimate tensile strength (MPa)	Maximum stress (MPa)	Yield status (N/Y)
H500-20	QT600	370/600	182.97	N
	QT550	350/550	182.97	N
	QT500	320/500	182.97	N
	QT450	310/450	182.97	N
	QT400 QT350	250/400 220/350	182.97 182.97	N N
H400-22	QT600	370/600	229.71	N
	QT550	350/550	229.71	N
	QT500	320/500	229.71	N
	QT450	310/450	229.71	N
	QT400 QT350	250/400 220/350	229.71 221.23	N Y
H400-20	QT600	370/600	222.69	N
	QT550	350/550	222.69	N
	QT500	320/500	222.69	N
	QT450	310/450	222.69	N
	QT400 QT350	250/400 220/350	222.69 221.04	N Y
H400-18	QT600	370/600	175.31	N
	QT550	350/550	175.31	N
	QT500	320/500	175.31	N
	QT450	310/450	175.31	N
	QT400 QT350	250/400 220/350	175.31 175.31	N N
H400-12	QT600	370/600	104.28	N
	QT550	350/550	104.28	N
	QT500	320/500	104.28	N
	QT450	310/450	104.28	N
	QT400 QT350	250/400 220/350	104.28 104.28	N N
H335-22	QT600	370/600	244.34	N
	QT550	350/550	244.34	N
	QT500	320/500	244.34	N
	QT450	310/450	244.34	N
	QT400 QT350	250/400 220/350	244.34 221.22	N Y
H335-20	QT600	370/600	241.91	N
	QT550	350/550	241.91	N
	QT500	320/500	241.91	N
	QT450	310/450	241.91	N
	QT400 QT350	250/400 220/350	241.91 221.13	N Y
H335-18	QT600	370/600	175.88	N
	QT550	350/550	175.88	N
	QT500	320/500	175.88	N
	QT450	310/450	175.88	N
	**QT400** **QT350**	**250/400** **220/350**	**175.88** **175.88**	**N** **N**

## References

[1] Bachmann H., Steinle A. (2011). Precast Concrete Structures.

[2] Huang Y., Zhu Z., Naito C.J., Yi W. (2017). Tensile nehavior of half grouted sleeve connections: Experimental study and analytical modeling. Constr. Build. Mater..

[3] Yee A. (1973). New precast prestressed system saves money in Hawaii hotel. PCI J..

[4] Einea A., Yamane T., Tadros M.K. (1995). Grout-filled pipe splices for precast concrete construction. PCI J..

[5] Lamport W., Jirsa J.O., Yura J.A. (1991). Strength and Behavior of Grouted Pile-To-Sleeve Connections. J. Struct. Eng..

[6] Arditi D., Ergin U., Gunhan S. (2000). Factors affecting the use of precast concrete systems. J. Archit. Eng..

[7] Zhao X., Ghojel J., Grundy P., Han L. (2006). Behaviour of grouted sleeve connections at elevated temperatures. Thin Walled Struct..

[8] Ling J.H., Rahman A.B.A., Ibrahim I.S., Hamid Z.A. (2016). Tensile capacity of grouted splice sleeves. Eng. Struct..

[9] Lin F., Wu X. (2016). Mechanical Performance and Stress–Strain Relationships for Grouted Splices under Tensile and Cyclic Loadings. Int. J. Concr. Struct. Mater..

[10] Zhu Z., Ahmad I., Mirmiran A. (2006). Splicing of Preacast Concrete-Filled FRP Tubes. J. Compos. Constr..

[11] Belleri A., Riva P. (2012). Seismic performance and retrofit of precast concrete grouted sleeve connections. PCI J..

[12] Popa V., Papurcu A., Cotofana D., Pascu R. (2015). Experimental testing on emulative connections for precast columns using grouted corrugated steel sleeves. Bull. Earthq. Eng..

[13] Ameli M.J., Pantelides C.P. (2017). Seismic Analysis of Precast Concrete Bridge Columns Connected with Grouted Splice Sleeve Connectors. J. Struct. Eng..

[14] Xu G., Wang Z., Wu B., Bursi O.S., Tan X., Yang Q., Wen L. (2017). Seismic performance of precast shear wall with sleeves connection based on experimental and numerical studies. Eng. Struct..

[15] Bathe K.J. (1982). Finite Element Procedures in Engineering Analysis.

[16] Mercan B., Schultz A.E., Stolarski H.K. (2010). Finite element modeling of prestressed concrete spandrel beams. Eng. Struct..

[17] Chou C.C., Chang H.J., Hewes J.T. (2013). Two-plastic-hinge and two dimensional finite element models for post-tensioned precast concrete segmental bridge columns. Eng. Struct..

[18] Zhao Z.Z., Zhou J., Hou J., Ren B. (2016). Finite element analysis of shear walls with precast concrete hollow moulds and splice rebar connection between the upper and lower floors. Eng. Mech..

[19] Pian T.H.H., Wu C.C. (2006). Hybrid and Incompatible Finite Element Methods.

[20] Wang H., Qin Q.H. (2017). Voronoi Polygonal Hybrid Finite Elements with Boundary Integrals for Plane Isotropic Elastic Problems. Int. J. Appl. Mech..

[21] Wang H., Qin Q.H., Xiao Y. (2016). Special n -sided Voronoi fiber/matrix elements for clustering thermal effect in natural-hemp-fiber-filled cement composites. Int. J. Heat Mass Transf..

[22] Wang H., Zhao X.J., Wang J.S. (2015). Interaction analysis of multiple coated fibers in cement composites by special n-sided interphase/fiber elements. Compos. Sci. Technol..

[23] Brebbia C.A. (1980). The Boundary Element Method for Engineers.

[24] Saleh A.L., Aliabadi M.H. (1995). Crack growth analysis in concrete using boundary element method. Eng. Fract. Mech..

[25] Lee C.Y., Wang H., Qin Q.H. (2016). Dual reciprocity boundary element method using compactly supported radial basis functions for 3D linear elasticity with body forces. Int. J. Mech. Mater. Des..

[26] Thanoon W.A., Alwathaf A.H., Noorzaei J., Jaafar M.S., Abdulkadir M.R. (2008). Nonlinear finite element analysis of grouted and ungrouted hollow interlocking mortarless block masonry system. Eng. Struct..

[27] Comite Euro-International Du Beton (1993). CEB-FIP Model Code 90 for Concrete Structures.

[28] American Concrete Institute (2014). Building Code Requirements for Structural Concrete (ACI 318-14).

[29] Ministry of Housing and Urban-Rural Development of the People’s Republic of China (2015). Technical Specification for Grout Sleeve Splicing of Rebars (JGJ355-2015).

[30] Crisfield M.A. (1991). Nonlinear Finite Element Analysis of Solids and Structures.

[31] Lu X.Z., Teng J.G., Ye L.P., Jiang J.J. (2005). Bond–slip models for FRP sheets/plates bonded to concrete. Eng. Struct..

[32] Hawileh R.A., Naser M.Z., Abdalla J.A. (2013). Finite element simulation of reinforced concrete beams externally strengthened with short-length CFRP plates. Compos. Part B-Eng..

[33] Naser M., Hawileh R., Rasheed H. (2014). Performance of RC T-Beams Externally Strengthened with CFRP Laminates under Elevated Temperatures. J. Struct. Fire Eng..

[34] Zhu B.L. (1991). Design Principle of Masonry Structure (in Chinese).

